# An Aza resveratrol–chalcone derivative 6b protects mice against diabetic cardiomyopathy by alleviating inflammation and oxidative stress

**DOI:** 10.1111/jcmm.13477

**Published:** 2018-01-12

**Authors:** Shengban You, Jianchang Qian, Chuchu Sun, Hailing Zhang, Shiju Ye, Taiwei Chen, Zheng Xu, Jingying Wang, Weijian Huang, Guang Liang

**Affiliations:** ^1^ Chemical Biology Research Center School of Pharmaceutical Sciences Wenzhou Medical University Wenzhou Zhejiang China; ^2^ Department of Cardiology the First Affiliated Hospital Wenzhou Medical University Wenzhou Zhejiang China; ^3^ Translational Medicine Center in Affiliated Yueqing Hospital Wenzhou Medical University Wenzhou Zhejiang China; ^4^ Department of Endocrinology the First Affiliated Hospital Wenzhou Medical University Wenzhou Zhejiang China

**Keywords:** inflammation, oxidative stress, diabetic cardiomyopathy, NF‐κB, Nrf2

## Abstract

Inflammation and oxidative stress play a crucial role in the development of diabetic cardiomyopathy (DCM). We previously had synthesized an Aza resveratrol–chalcone derivative 6b, of which effectively suppressing lipopolysaccharide (LPS)‐induced inflammatory response in macrophages. This study aimed to investigate the potential protective effect of 6b on DCM and underlying mechanism. In H9c2 myocardial cells, 6b potently decreased high glucose (HG)‐induced cell fibrosis, hypertrophy and apoptosis, alleviating inflammatory response and oxidant stress. In STZ‐induced type 1 diabetic mice (STZ‐DM1), orally administration with 6b for 16 weeks significantly attenuated cardiac hypertrophy, apoptosis and fibrosis. The expression of inflammatory cytokines and oxidative stress biomarkers was also suppressed by 6b distinctly, without affecting blood glucose and body weight. The anti‐inflammatory and antioxidative activities of 6b were mechanistic associated with nuclear factor‐kappa B (NF‐κB) nucleus entry blockage and Nrf2 activation both *in vitro* and *in vivo*. The results indicated that 6b can be a promising cardioprotective agent in treatment of DCM *via* inhibiting inflammation and alleviating oxidative stress. This study also validated the important role of NF‐κB and Nrf2 taken in the pathogenesis of DCM, which could be therapeutic targets for diabetic comorbidities.

## Introduction

Cardiovascular disease is considered to be a leading cause of morbidity and mortality in diabetic patients 1. DCM is characterized by structural and functional abnormalities in the myocardium such as cardiac fibrosis, ventricular hypertrophy and heart failure 2. Several mechanisms have been implicated to be associated with the pathogenesis of DCM, including myocardial insulin resistance, cardiac inflammation, oxidative stress, interstitial fibrosis, cardiac cell death and apoptosis 3, 4, 5, 6.

It is well known that hyperglycaemia, the major clinical presentation of diabetes, is associated with chronic inflammation and oxidative stress 7. Hyperglycaemia can increase the expression and release of pro‐inflammatory cytokines such as interleukin 6 (IL‐6), IL‐1b and tumour necrosis factor α (TNF‐α), by activating the NF‐κB pathway 8, 9. For another, hyperglycaemia can also increase reactive oxygen species (ROS) and/or reactive nitrogen species (RNS) production from both non‐mitochondrial and mitochondrial sources 10. The crosstalk between inflammation and oxidative stress signalling can further influence on each other. Thus, inhibition of inflammation and oxidative stress could be promising strategy for treatment of DCM.

**Figure 1:**
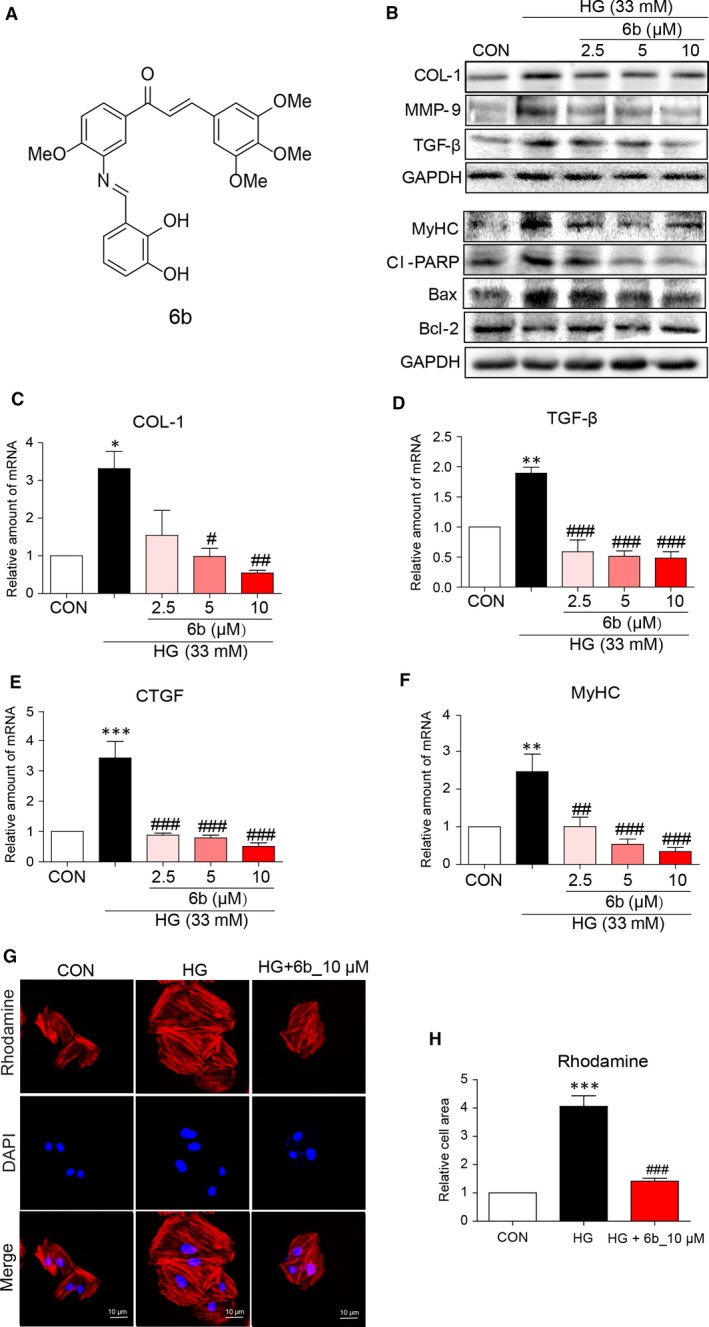
6b attenuates HG‐induced cardiac hypertrophy, fibrosis and apoptosis in H9C2 cells. **A**. The chemical structures of Aza resveratrol–chalcone derivative 6b. **B**. H9c2 cells were pre‐treated with indicated dose of 6b for 1 hr and then incubated with HG (33 mM) for 36 hrs. Cells were harvested, and total lysates were immunoblotted. **C–F**. H9c2 cells were pre‐treated with indicated dose of 6b for 1 hr, following HG (33 m) incubation for 12 hrs. mRNA was extracted and subjected to real‐time qPCR assay as described in Materials and methods. Normalized values are plotted for COL‐1, TGF‐β, CTGF and MyHC, respectively. **G** and **H**. Rhodamine–phalloidin staining assay was performed as described in Materials and methods. The images were quantified (**G**). Normalized values are plotted (**H**). *P < 0.05, **P < 0.01, ***P < 0.001 *versus* CON; ^#^ *P* < 0.05, ^##^ *P* < 0.01, ^###^ *P* < 0.001 *versus* HG.

The discovery of natural products may help the identification of bioactive lead compounds with anti‐inflammatory and antioxidative stress properties. Aza resveratrol–chalcone, considered as the precursors of resveratrol and chalcone, is abundant in edible plant. Resveratrol and chalcone have been reported to possess a diverse variety of pharmacodynamic effects on vascular diseases, cancer, viral infections and inflammation, both *in vitro* and *in vivo* 11, 12. Considered Pingaew *et al*.'s strategy to derive a series of chalcone–coumarin hybrids (anticancer and antimalarial agents), we had designed and synthesized a series of Aza resveratrol–chalcone derivatives with enhanced anti‐inflammatory activity and reduce cytotoxicity 13. Among them, 6b (structure illustrated in Fig. 1A) has the highest potency on the suppression of LPS‐induced inflammation 13. Therefore, we hypothesized that 6b would be able to attenuate HG‐induced inflammation and oxidant stress. In this study, we investigated pharmacodynamic effect of 6b on DCM model as well as underlying mechanism both *in vitro* and *in vivo*.

## Materials and methods

### Reagents

Compound 6b was synthesized by our group and prepared with a purity of 98.9% as described previously 11. The chemical structure is shown in Figure 1A. 6b was dissolved in dimethyl sulfoxide (DMSO) as 20 mM stock solution and diluted before assays. The final concentration of DMSO was 0.1% (v/v). All other chemicals were purchased from Sigma‐Aldrich unless otherwise specified.

### Cell culture and treatment

H9c2, a embryonic rat heart‐derived cell line, was obtained from the Shanghai Institute of Biochemistry and Cell Biology (Shanghai, China) and cultured in DMEM medium (Gibco, Eggenstein, Germany) with 5.5 mM D‐glucose, 10% FBS, 100 U/ml of penicillin and 100 mg/ml of streptomycin. Cells were pre‐treated with or without 6b for 1 hr and then exposed to HG (33 mM) DMEM medium for different time.

### Cell viability and secretion of TNF‐α determination

Seed cells into 96‐well plates before treatment, 8 × 10^3^ cells/well. Add drug into wells with various dose and incubated for 48 hrs. After treatment, MTT was added to each well (1 mg/ml), incubated 37°C for 4 hrs. The formazan crystal was dissolved with DMSO, 150 μl/well. The absorbance was detected at 490 nm on a microplate reader. Cell viability was expressed as the percentage of MTT reduction compared to control.

TNF‐α in cell culture medium was measured using ELISA kit (eBioscience, San Diego, CA, USA). The total amount of TNF‐α was normalized to the total amount of protein in the viable cell pellets.

### Animal experiments

The animals were obtained from Animal Center of Wenzhou Medical University. Protocols used for all animal studies were approved by the Wenzhou Medical University Animal Policy and Welfare Committee and complied with the NIH guidelines (Guide for the care and use of laboratory animals). Male C57BL/6 mice, weighing 23–25 g, were used for study. Mice were received intraperitoneal (i.p.) injection of streptozotocin (STZ) at the dose of 50 mg/kg formulated in 100 mM citrate buffer (pH 4.5) for 5 consecutive days 1 week later, blood glucose levels were detected utilizing a glucometer. The compound treatment was initiated after the establishment of type 1 diabetes mellitus (fasting blood glucose >12 mM). STZ‐induced diabetic mice (STZ‐DM1) were orally treated with 6b (5 or 20 mg/kg) or vehicle (1% CMC‐Na) by gavage, qod, for 16 weeks (*n* = 9, each group). At the indicated time‐points, blood glucose and body weight were recorded, Figure S1A. Sixteen weeks after the administration, mice were terminated under anaesthesia by 100 mg/kg body weight ketamine hydrochloride (Ketanest, Pfizer, Germany) and 16 mg/kg body weight xylazine hydrochloride (Rompun 2%, Bayer, Leverkusen, Germany). Blood samples were collected and centrifuged at 1500 g, 4°C for 10 min. to collect the serum. Heart tissues were embedded in 4% paraformaldehyde or snap‐frozen in liquid nitrogen for further analysis.

### Cardiac function detection

Systolic and diastolic cardiac function was determined non‐invasively by transthoracic echocardiography in anaesthetized mice 1 day before termination 10. Acuson‐sequoia 512 was used and equipped with an acuson‐15L8w probe at the frequency of 12–14 MHz. The image acquired in the M‐mode and short‐axis. Diastolic function was assessed using pulsed‐wave Doppler imaging of the transmitral filling pattern. Ejection fraction (EF) was calculated from LV end‐diastolic volume (LVEDV) and end‐systolic volume (LVESD) using the equation of (LVEDV − LVESV)/LVEDV × 100%. Fractional shortening (FS) was calculated using the equation (FS = [(LVIDd − LVIDs)/LVIDd] × 100%). Tei index was determined based on Doppler recordings of left ventricular (LV) isovolumetric relaxation time (IRT), isovolumetric contraction time (ICT) and ejection time (ET) [Tei = (IRT + ICT)/ET].

### Determination of serum CK‐MB and insulin levels

Serum levels of insulin and creatine kinase (CK‐MB) were analysed by commercial ELISA kits refer to the manufacturers' instruction (BIOSINO Bio‐tech Inc., Shanghai, China).

### Real‐time quantitative PCR

Cells or heart tissues were homogenized in TRIZOL (Thermo Fisher, Shanghai, China). RNA was extracted following the procedure of standard protocol. Reverse transcription and quantitative PCR were carried out using two‐step M‐MLV Platinum SYBR Green qPCR SuperMix‐UDG kit (Thermo Fisher) and Eppendorf Mastercycler ep realplex detection system (Eppendorf, Hamburg, Germany). Primers were synthesized and obtained from Thermo Fisher, Table S1.

### Histomorphology and immunohistochemistry examination

Hearts of mice were fixed in 4% paraformaldehyde and embedded. The paraffin of each sample was sectioned at 5 μm thick and performed haematoxylin and eosin (H&E), Sirius red and Masson trichrome staining as standard protocols. For immunohistochemical staining, tissue sections were deparaffinized with xylene, rehydrated in graded alcohol series, subjected to antigen retrieval in 0.01 M citrate buffer (pH 6.0) by microwaving and then placed in 3% hydrogen peroxide in methanol for 30 min. at room temperature. Slides were blocked with 1% BSA in PBS for 30 min. and then incubated with primary antibody at 4°C overnight (TNF‐a, 1:100; VCAM, 1:100; CD68, 1:100; 3‐NT, 1:100; P65, 1:200). Peroxidase‐conjugated secondary antibodies were used follow‐on (Santa Cruz, Dallas, Texas, USA, 1:100 dilution; 1 hr incubation). Finally, slides were counterstained with haematoxylin for 5 min., dehydrated and mounted. Images were viewed and acquired by microscope (Nikon, Shinagawa, Tokyo, Japan).

TUNEL staining was performed with the terminal deoxynucleotidyl transferase‐mediated dUTP nick end labelling (TUNEL) apoptosis detection kit (R&D Systems, Minneapolis, MN, USA). Seed 10^5^ H9c2 cells in a 30‐mm cell culture dish. After treatment, cells were fixed with 4% paraformaldehyde, permeabilized with 0.1% Triton X‐100 and stained with rhodamine–phalloidin at a concentration of 50 μg/ml for 30 min. Nuclei were stained with the DAPI at room temperature. Images were captured and quantified by ImageJ (https://imagej.nih.gov/ij/). The cell area measurements were normalized by the number of cells analysed in the sight of 400× views. For P65, Nrf2 and NF‐κB determination, cells were incubated with anti‐P65 antibody (1:200) after fixation overnight at 4°C. PE‐conjugated secondary antibody (1:200) was used and followed. Fluorescent images were viewed and captured by fluorescence microscope (Nikon).

### Determination of Intracellular ROS

Hearts were fixed in 4% paraformaldehyde. The sections were prepared as described in ‘2.8’. Slides were incubated in PBS containing 2 μM DHE for 2 hrs at 37°C and washed three times. Immunofluorescence image was captured and measured using Nikon fluorescence microscope at excitation wavelengths of 535 or 488 nm (Nikon).

### Immunoblotting

Cells or tissues (30–50 mg) were homogenized and lysed with lysis buffer (AR0101/0103, Boster Biological Technology Co. Ltd, Pleasanton, CA, USA). Lysates and nuclear extract (P00028; Beyotime, Beijing, China) were separated by SDS‐PAGE gel and electrotransferred to polyvinylidene fluoride membranes. The membranes were blocked for 1.5 hr at room temperature in Tris‐buffered saline (TBS), pH 7.6, containing 0.05% Tween 20 and 5% non‐fat milk. Myosin heavy chain (MyHC, sc‐20641), B‐cell lymphoma‐2 (Bcl‐2, sc‐492), BCL2‐associated X (Bax, sc‐492), cleaved poly ADP‐ribose polymerase (CL‐PARP, sc‐56196), collagen 1 (COL‐1, sc‐59772), matrix metalloproteinase 9 (MMP‐9, sc‐6841), transforming growth factor‐β (TGF‐β, sc‐146), NF‐κB P65 (sc‐7151), nuclear factor (erythroid‐derived 2)‐like 2 (Nrf2, sc‐13032), haem oxygenase 1 (HO‐1, sc‐136256), NAD(P)H quinone dehydrogenase 1 (NQO‐1, sc‐271116) and Lamin B (sc‐56143) were purchased from Santa Cruz (Dallas, Texas, USA). Monocyte–macrophage glycoprotein CD68 (ab‐955), vascular cell adhesion molecule 1 (VCAM‐1, ab‐134047), tumour necrosis factor‐α (TNF‐α, ab‐1392), 3‐nitrotyrosine (3‐NT, ab‐61392) and glyceraldehyde‐3‐phosphate dehydrogenase (GAPDH, ab‐8245) antibodies were obtained from Abcam (Cambridge, MA, USA). Primary and second antibodies were diluted in TBS buffer (containing 0.05% Tween 20). Specific band was detected by incubation of enhanced chemiluminescence kit (Bio‐Rad, Hercules, CA, USA).

### Statistical analysis

All data represent three independent experiments and are expressed as mean ± S.E.M. All statistical analyses were performed with GraphPad Pro Prism 5.01 (GraphPad, San Diego, CA, USA). One‐way ANOVA followed by multiple comparisons test with Bonferroni correction was employed to analyse the differences between sets of data. *P* value <0.05 was considered significant.

## Results

### 6b attenuated HG‐induced cell fibrosis, hypertrophy and apoptosis in H9c2 cells

To investigate the effect of 6b on cell proliferation, H9c2 cells were treated with several doses of 6b for 48 hrs. Cell viability was evaluated by MTT assay. As Figure S2 shown, there is no significant inhibition. The biomarkers of cell fibrosis, hypertrophy and apoptosis were assessed by immunoblotting after treatment, Figure 1B. 6b significantly inhibited the expression of pro‐fibrotic markers that stimulated by HG, including COL‐1, MMP‐9 and TGF‐β, Figure 1B. It is associated with dose‐dependent suppression of transcription level of COL‐1 and TGF‐β, as well as connective tissue growth factor (CTGF), Figure 1C–E. The cell size of H9c2 that incubated with HG was fourfold as large as control (CON). However, cell enlargement was significantly prevented by 6b, Figure 1G–H. Concomitantly, MyHC, a hypertrophy biomarker, was dose‐dependently inhibited by 6b comparing to HG‐stimulated cells both at protein and transcription levels, Figure 1B and F. Moreover, apoptotic biomarker CL‐PARP was significantly down‐regulated by 6b under HG challenge, consistent with normalized Bcl‐2, Figure 1B.

### 6b mitigated NF‐κB‐mediated inflammatory response in HG‐stimulated H9c2 cells

**Figure 2:**
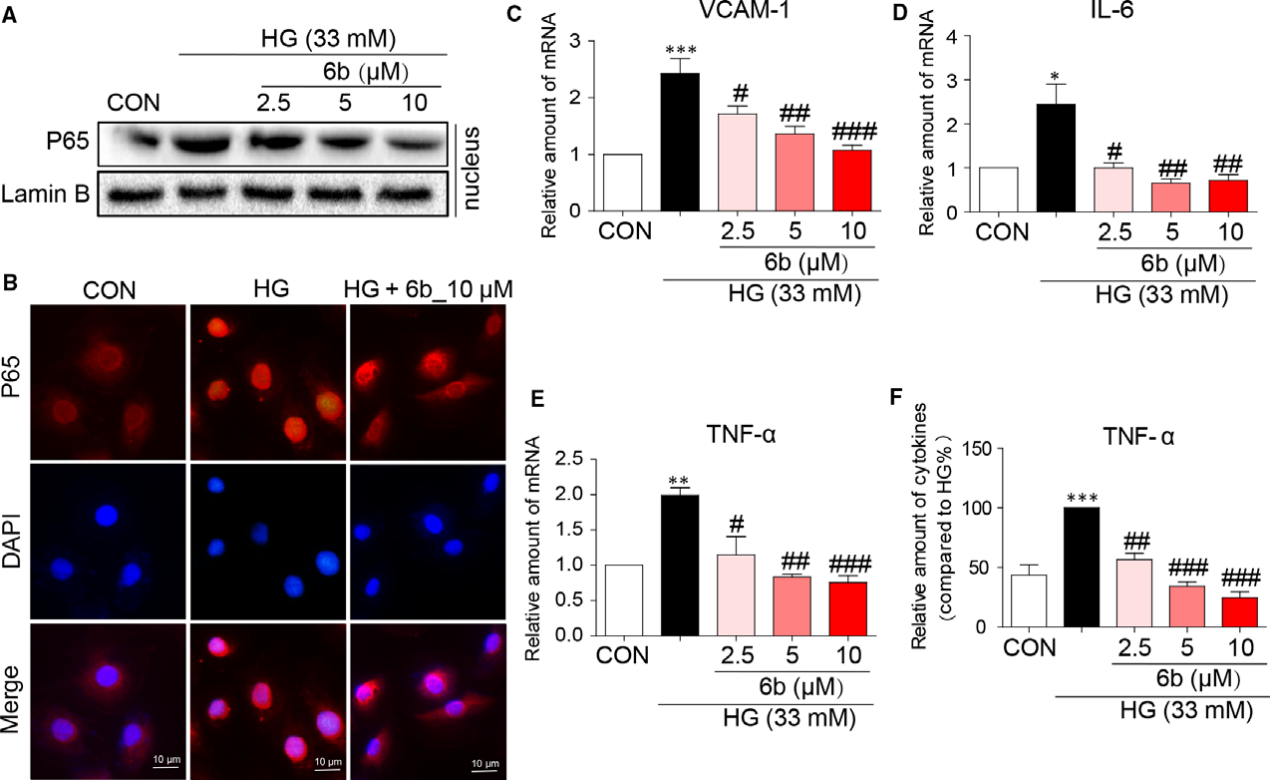
6b mitigates HG‐induced NF‐κB‐mediated inflammatory response in H9c2 cells. A–B. H9c2 cells were pre‐treated with indicated dose of 6b for 1 hr and then incubated with HG (33 mM) for 8 hrs. The cell lysates were made, following nucleus protein extraction and immunoblotted (**A**). Immunofluorescence assay was performed as described in Materials and methods (**B**). The mRNA expression levels of VCAM‐1 (**C**), IL‐6 (**D**) and TNF‐α (**E**) were determined. The normalized values are plotted. **F**. H9c2 cells were pre‐treated with 6b for 1 hr, incubated with HG (33 mM) for 24 hrs as followed. TNF‐α secretion was subjected to ELISA as described in Materials and methods. Values detected were normalized with total protein. *P < 0.05, **P < 0.01, ***P < 0.001 *versus* CON; ^#^ *P* < 0.05, ^##^ *P* < 0.01, ^###^ *P* < 0.001 *versus* HG.

To confirm the pharmacological effects of 6b on HG‐induced H9c2 cells were associated with anti‐inflammatory, cell nucleus fraction was extracted and immunoblotted for NF‐κB. P65 in nucleus was obviously suppressed by 6b under HG condition, Figure 2A. Consistently, immunostaining data directly showed less positive staining of P65 in the nucleus compared 6b pre‐treatment group to HG, Figures 2B and S4. In addition, the mRNA expression of downstream pro‐inflammatory cytokines including VCAM, IL‐6 and TNF‐α was significantly decreased after treatment, Figure 2C–E. In consistence with inhibition of TNF‐α transcription level, TNF‐α secretion was also dose‐dependently inhibited by 6b, Figure 2F. These data indicated that 6b significantly reduced HG‐induced pro‐inflammatory cytokine expression by preventing the nuclear translocation of P65 NF‐κB.

### 6b inhibited HG‐induced oxidative stress *via* augmenting antioxidant Nrf2 pathway in H9c2 cells

**Figure 3:**
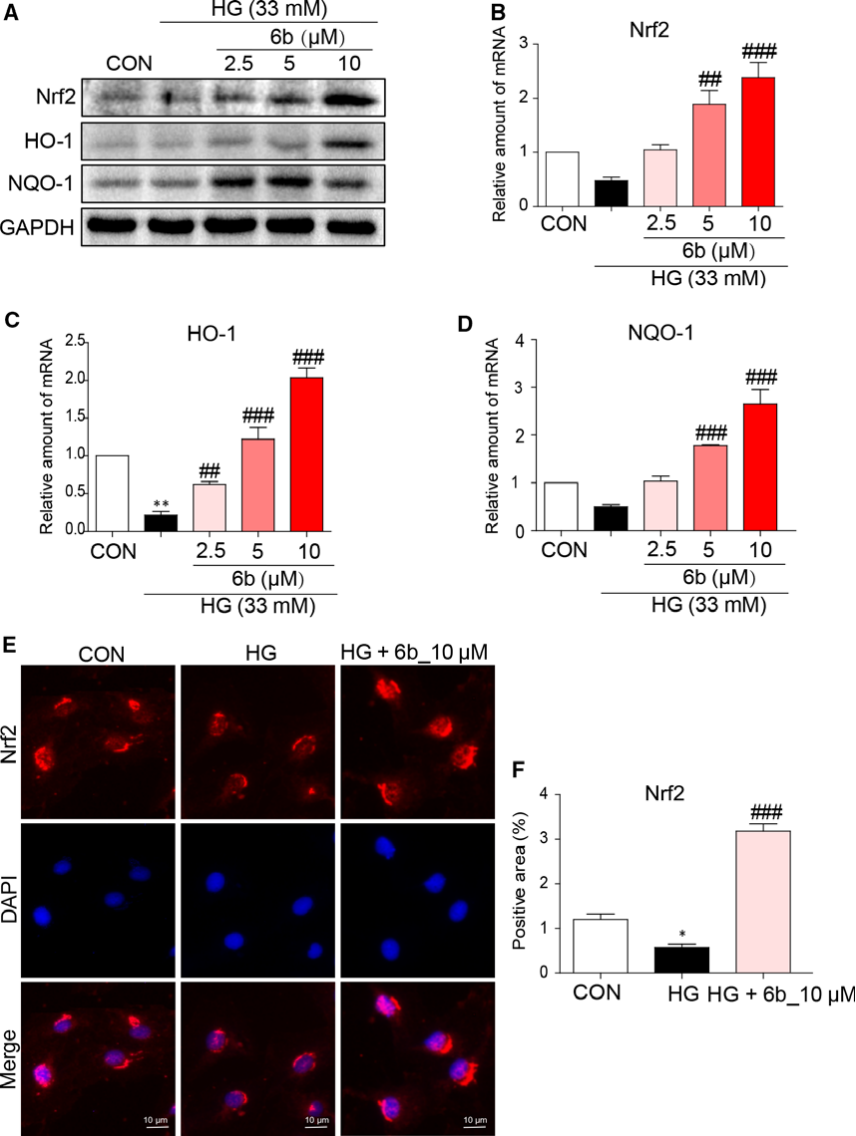
6b attenuates HG‐induced myocardial oxidative stress in H9c2 cells. H9c2 cells were pre‐treated with indicated dose of 6b for 1 hr and then incubated with HG (33 mM) for 12 hrs. The cell lysates were immunoblotted for Nrf2, HO‐1 and NQO‐1, respectively (**A**). The mRNA expression levels of Nrf2 (**B**), NQO‐1 (**C**) and HO‐1 (**D**) were determined. Cells were subjected to immunofluorescence assay as described in Materials and methods. Images were acquired (**E**) and quantified (**F**). Normalized values are plotted. *P < 0.05, **P < 0.01 *versus* CON; ^##^ *P* < 0.01, ^###^ *P* < 0.001 *versus* HG.

Antioxidant effect of 6b could also contribute to DCM prevention. Therefore, H9c2 cells that being stimulated by HG were immunoblotted, Figure 3A. The cellular redox level and detoxification response are mainly mediated by Nrf2, a transcription factor that regulates the gene expression of antioxidant enzymes 14. 6b significantly up‐regulated Nrf2 as well as downstream antioxidant genes, including HO‐1 and NADPH quinine oxidoreductase (NQO‐1). It is correlated with suppression of transcription levels of Nrf2, HO‐1 and NQO‐1, Figure 3B–D. Furthermore, immunostaining of Nrf2 further proved that 6b maintained the high expression level of Nrf2 in cell nucleus in response to HG challenge (Fig. 3E–F). The data indicated that the antioxidative properties of 6b also contribute to its cardioprotective effect during HG challenge.

### 6b alleviated cardiac hypertrophy, apoptosis and fibrosis in the diabetic myocardium

**Figure 4:**
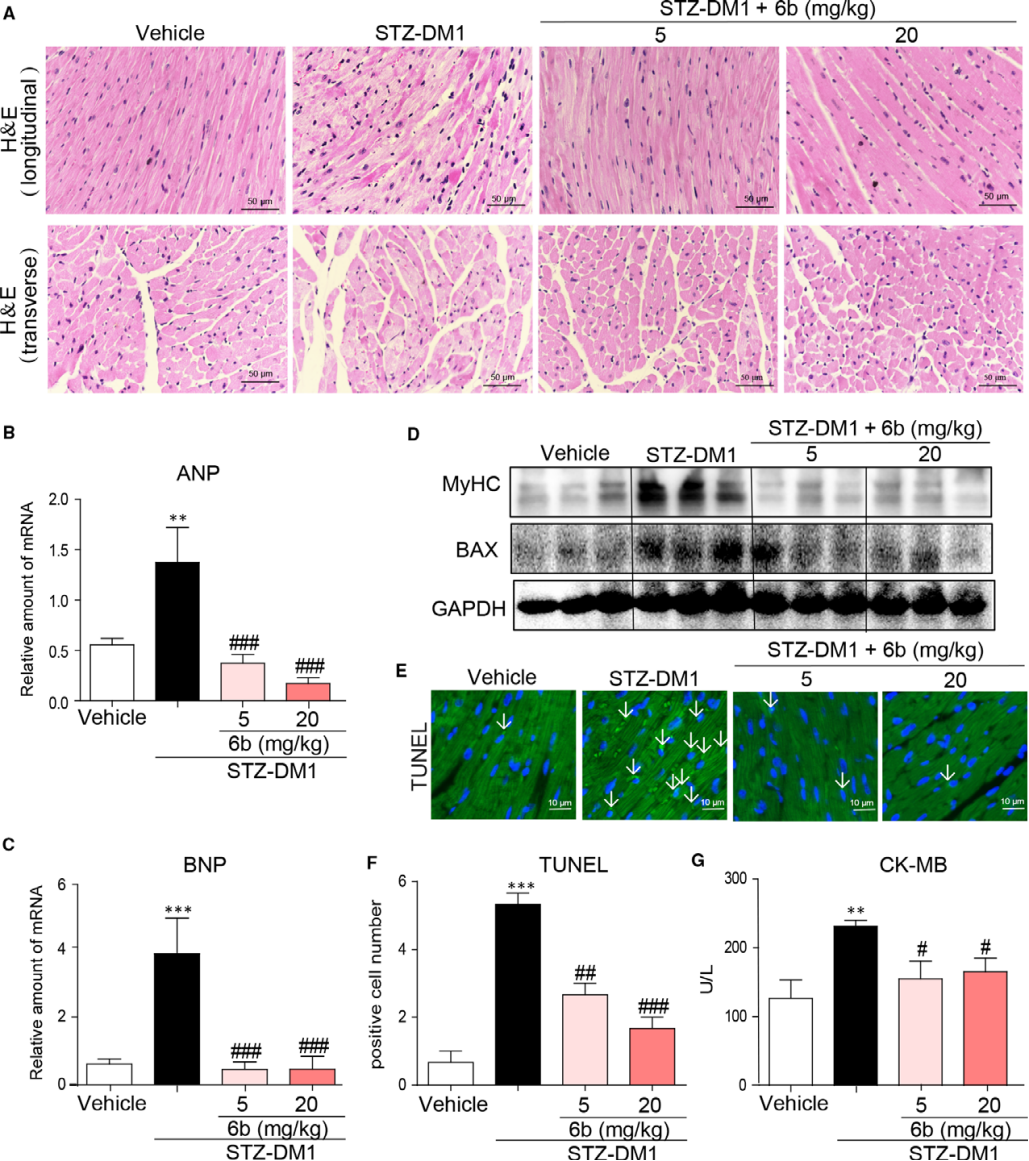
6b mitigates cardiac hypertrophy and apoptosis in diabetic myocardium. **A**. Representative histomorphometric images for the haematoxylin and eosin staining (H&E) of formalin‐fixed myocardial tissues from each group (400× magnification). **B** and **C**. The mRNA expression levels of ANP and BNP were subjected to real‐time qPCR assay as described in Materials and methods. **D**. Total tissue lysates were immunoblotted with anti‐MyHC and anti‐BAX antibodies. **E** and **F**. Heart tissues were subjected to TUNEL assay as described in Materials and methods. TUNEL images (400× magnification) were acquired (**E**) and quantified (**F**). **G**. Serum CK‐MB was determined as described in Materials and methods. **P < 0.001 *versus* Vehicle; ^#^ *P* < 0.05, ^##^ *P* < 0.01, ^###^ *P* < 0.001 *versus* STZ‐DM1.

DCM is characterized by LV hypertrophy and reduced cardiac function 15. STZ‐DM1 were employed to investigate the effect of 6b on DCM *in vivo*. In STZ‐DM1, myocardial tissues displayed structural abnormalities, but significantly improved by 6b treatment, Figures 4A and S3. Consistent with the histomorphometric observation, the transcription levels of ANP, BNP and MyHC were decreased by 6b, Figure 4B–C. In addition, the intraventricular septum and left ventricle wall were elevated, while EF and FS were significantly dropped in STZ‐DM1. However, 6b potently improved cardiac structure abnormalized as demonstrated by echocardiographic assessment, Table 1. Moreover, 6b has beneficial impact on survival of cardiomyocyte in DCM. As shown in Figure 4D–G, 6b treatment significantly down‐regulated pro‐apoptotic protein Bax, leading to less cell apoptosis (reduced positive TUNEL staining area) and cardiac muscle injury (assessed by serum creatinine).

**Table 1:** Standard echocardiographic data of each group

	Vehicle	STZ‐DM1	STZ‐DM1 + 6b_5 mg/kg	STZ‐DM1 + 6b_20 mg/kg
HR, bpm	428 ± 51	452 ± 75	493 ± 37	501 ± 53
HW/BW, mg/g	6.43 ± 0.34	7.39 ± 0.71*	6.43 ± 0.52	6.40 ± 0.43#
IVsd, mm	7.70 ± 0.14	8.13 ± 0.15*	7.74 ± 2.67#	7.51 ± 0.09###
LVIDd, mm	23.00 ± 0.82	26.676 ± 1.21***	23.57 ± 1.72###	23.29 ± 0.76###
IRT, ms	18.15 ± 4.02	32 ± 9.76***	20.75 ± 3.85##	17.67 ± 5.50###
Tei index	0.59 ± 0.13	1.01 ± 0.10***	0.78 ± 0.13#	0.70 ± 0.08###
EF%	81.52 ± 2.07	74.83 ± 2.93***	79.30 ± 3.43#	80.38 ± 0.48#
FS%	44.39 ± 1.76	37.47 ± 2.55***	42.42 ± 3.54#	43.90 ± 2.30#

HR, heart rate; HW/BW, heart weight/body weight; IVsd, diastolic interventricular septal thickness; LVIDd, left ventricular internal diameter at end‐diastole; IRT, isovolumic relaxation time; EF%, ejection fraction; FS%, fraction shortening.

*P < 0.05, ***P < 0.001 *versus* Vehicle; ^#^ *P* < 0.05, ^##^ *P* < 0.001, ^###^ *P* < 0.001 *versus* STZ‐DM1.

**Figure 5:**
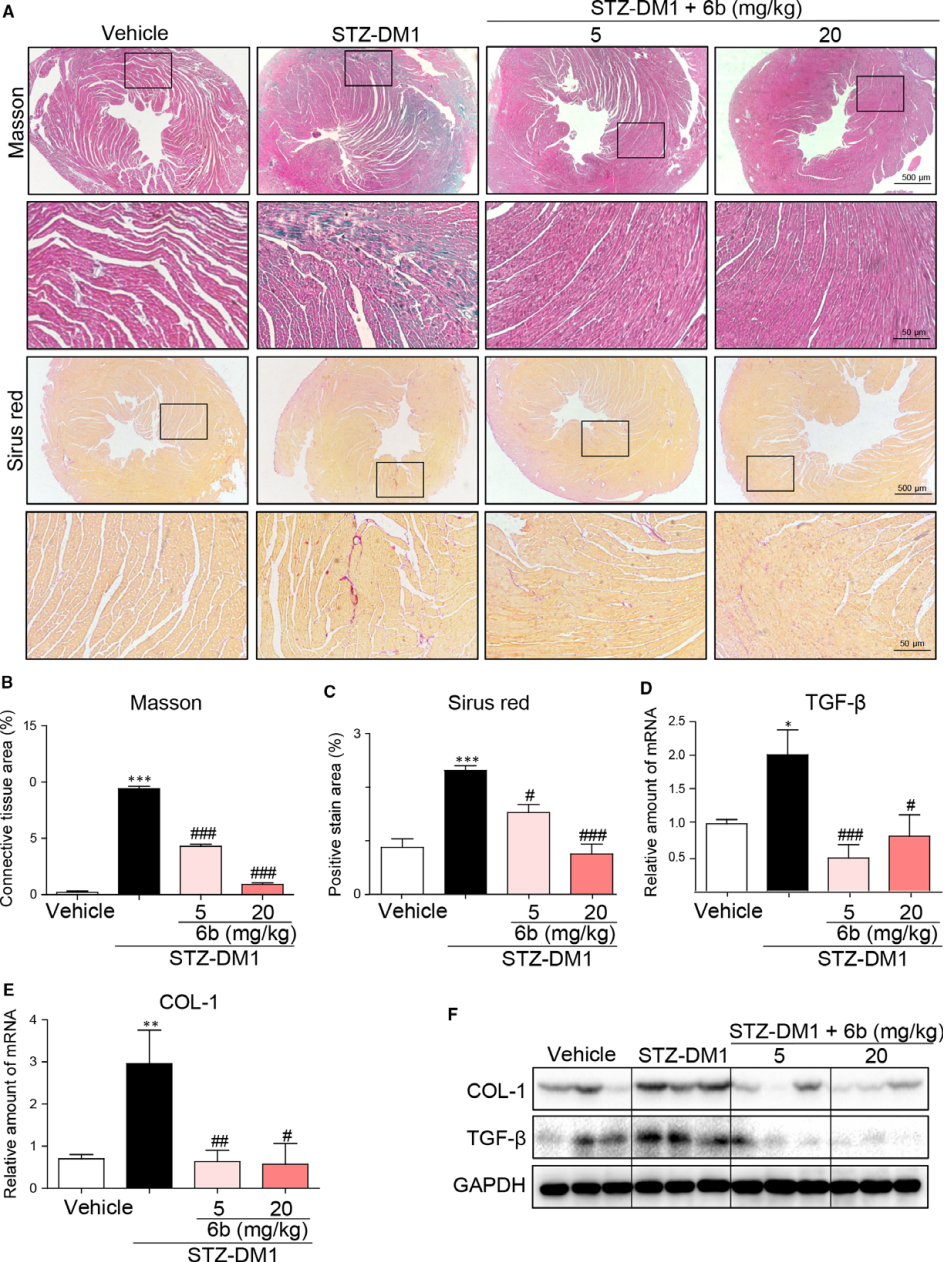
6b attenuates diabetes‐induced myocardial fibrosis. **A–C**. Myocardial tissues from each group were subjected to Masson Trichrome‐ and Sirius red staining assay as described in Materials and methods. Images were acquired (**A**) and quantified accordingly (**B** and **C**). **D–E**. The mRNA expression levels of TGF‐β and collagen I (COL‐1) in myocardial tissues of each group were determined by real‐time qPCR. **F**. The heart tissue lysates were immunoblotted with anti‐TGF‐β and anti‐COL‐1 antibodies, respectively. *P < 0.05, **P < 0.01, ***P < 0.001 *versus* Vehicle; ^#^ *P* < 0.05, ^##^ *P* < 0.01, ^###^ *P* < 0.001 *versus* STZ‐DM1.

6b also exerted an antifibrotic property in the DCM. Masson trichrome staining and Sirius red staining revealed a marked interstitial collagen accumulation in the heart of STZ‐DM1, collagen deposition was significantly reduced after being treated, Figure 5A–C. Consistent with the evidence from the microscopic examination of the cardiac tissue sections, pro‐fibrotic markers TGF‐β and Col‐1 were both normalized by 6b treatment at transcription and protein levels, Figure 5D–F. However, these beneficial effects of 6b on cardiac morphology and function might not due to any metabolic changes. As shown in Figure S1B‐D, 6b treatment did not affect fasting glucose, body weight and serum insulin.

### 6b attenuated diabetes‐induced myocardial inflammation and oxidative stress

**Figure 6:**
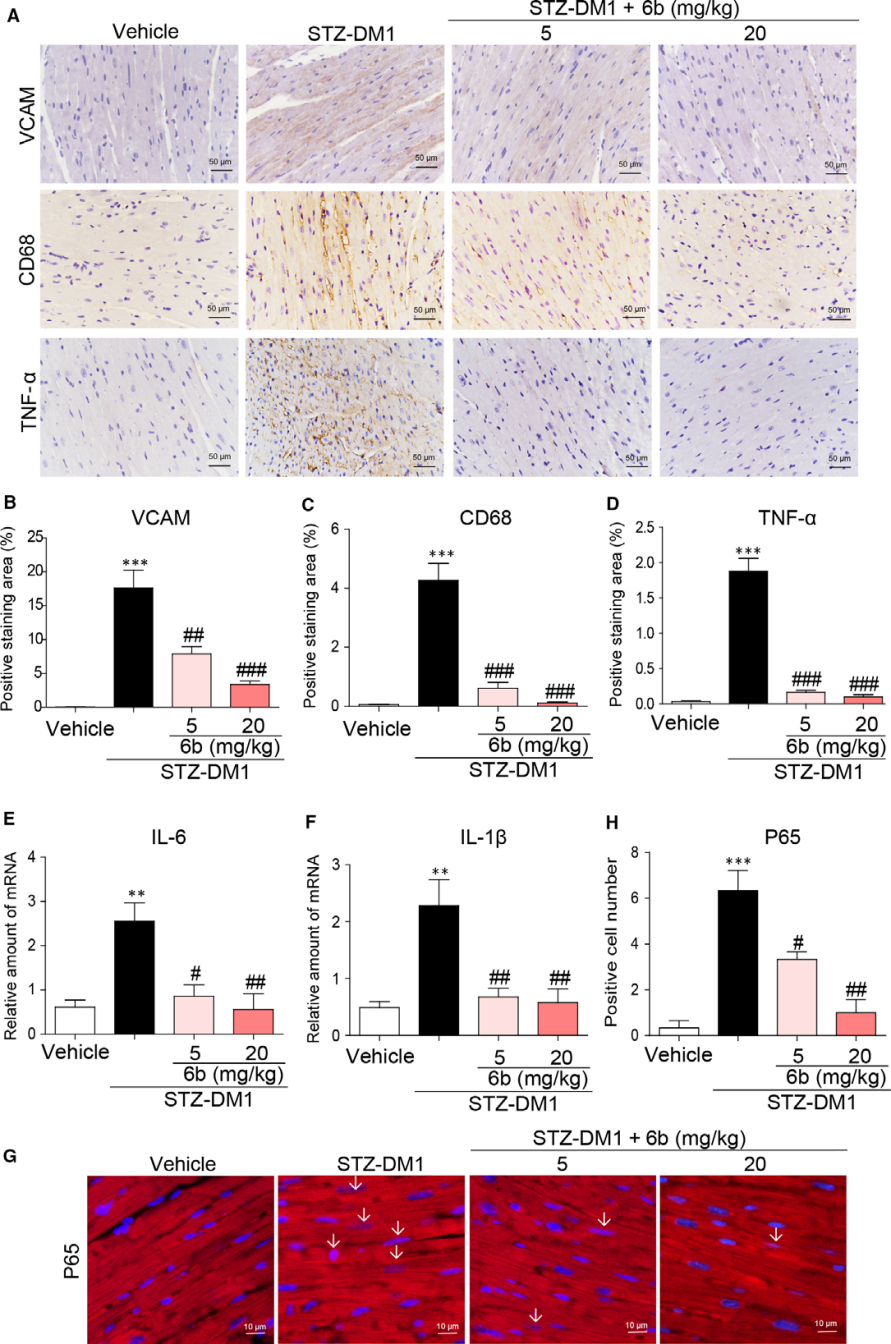
6b inhibits NFκB‐mediated myocardial inflammation in diabetic myocardium. **A–D**. Heart tissues were subjected to IHC analysis with anti‐VCAM‐1, anti‐CD68 and anti‐TNF‐α antibodies as described in Materials and methods (400× magnification). Images were acquired (**A**) and quantified accordingly (**B–D**). **E** and **F**. The mRNA expression levels of IL‐6 and IL‐1β in myocardial tissues were determined using real‐time qPCR. **G** and **H**. Heart tissues were subjected to immunofluorescence with anti‐P65 antibody as described in Materials and methods (400 × magnification). The images were quantified, and the normalized values are plotted (**H**). **P < 0.01, ***P < 0.001 *versus* Vehicle; ^#^ *P* < 0.05, ^##^ *P* < 0.01, ^###^ *P* < 0.001 *versus* STZ‐DM1.

To confirm the molecular mechanism underlying the protective effect of 6b *in vivo*, we examined the inflammatory and oxidative indexes in DCM with or without 6b administration. Macrophage infiltration was significantly inhibited by 6b as reduced VCAM and CD68‐positive staining, Figure 6A–C. Meanwhile, inflammatory cytokines TNF‐α, IL‐6 and IL‐1β were obviously inhibited, Figure 6A and D–F.

It is well defined that HG induces pro‐inflammatory cytokine expression through NF‐κB signalling pathway 12. Thus, we studied the impact of 6b on the NF‐κB (P65) activation. The immunostaining of P65 showed more positive cells in STZ‐DM1, while the number of these cells was dramatically decreased with 6b treatment, Figure 6G–H.

**Figure 7:**
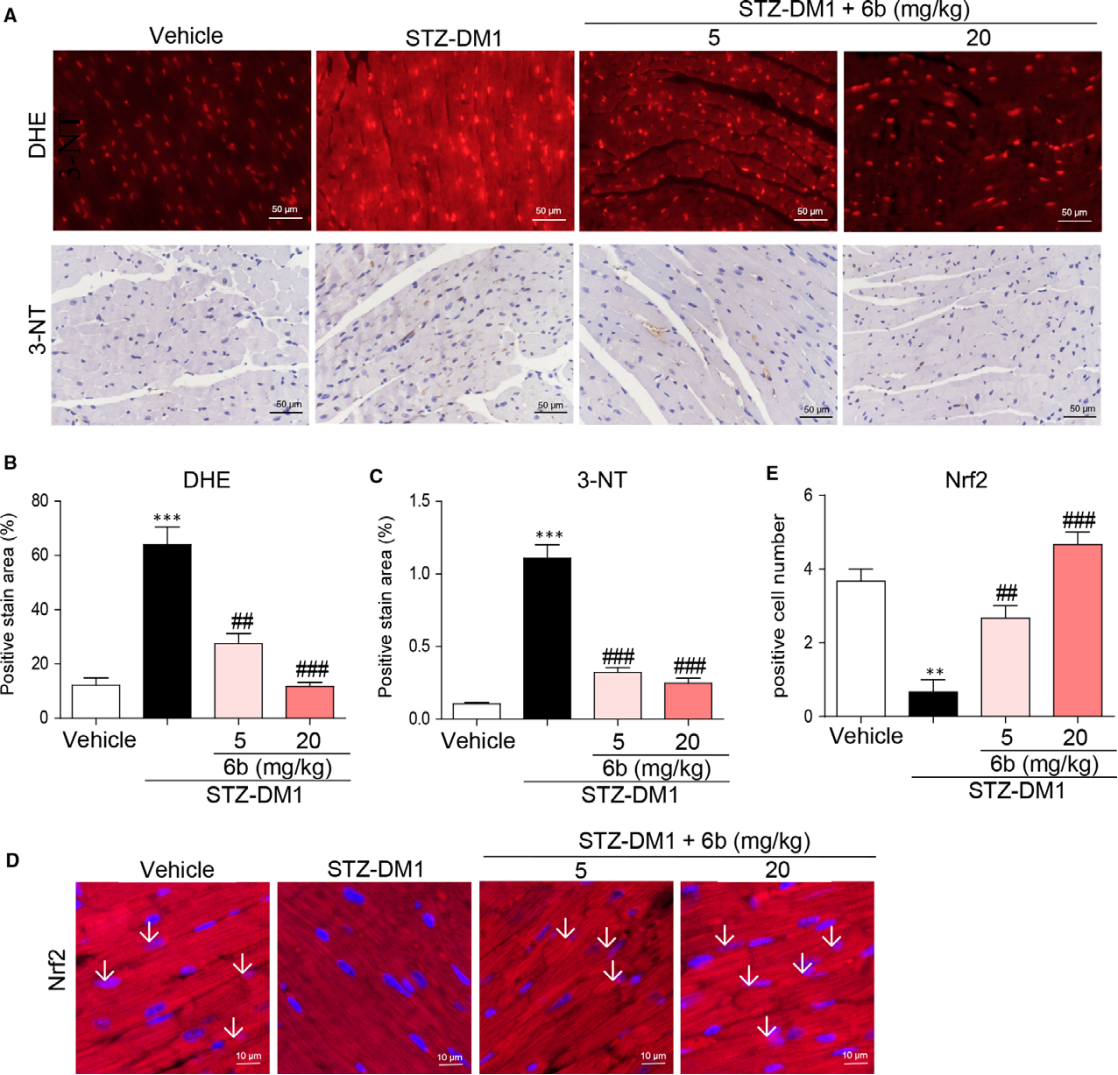
6b attenuates diabetes‐induced myocardial oxidative mechanistic through Nrf2. **A–C**. Myocardial tissues from STZ‐induced diabetic mice were subjected to immunostaining with anti‐DHE and anti 3‐NT antibodies as described in Materials and methods. The images were acquired (**A**) and quantified (**B** and **C**). **D** and **E**. Heart tissues from STZ‐induced diabetic mice were subjected to immunofluorescence with anti‐Nrf2 antibody. The images were acquired (**D**) and quantified (**E**). **P < 0.01, ***P < 0.001 *versus* Vehicle; ^##^ *P* < 0.01, ^###^ *P* < 0.001 *versus* STZ‐DM1.

We further detected the antioxidative effects of 6b in diabetic hearts. Quantification of oxidative stress in the myocardium through immunostaining with DHE and 3‐NT revealed lower levels of superoxide in 6b‐treated mouse hearts compared to STZ‐DM1, Figure 7A–C. The immunostaining showed that the Nrf2‐positive staining was much more in vehicle than STZ‐DM1; however, 6b treatment normalized or increased the nuclear localization of Nrf2, Figure 7D and E.

## Discussion

Recent studies have implicated that chronic inflammation and oxidative stress play a vital role in the pathophysiology of hyperglycaemia‐induced cardiovascular disorder. Increased production of inflammatory cytokines and ROS due to hyperglycaemia impairs normal cellular functions and causes apoptosis of cardiomyocytes 16, 17. Therefore, target of inflammation and oxidative stress during disease progression could be a potential therapeutic option for the treatment of diabetes and hyperglycaemia‐induced cardiovascular disorders.

**Figure 8:**
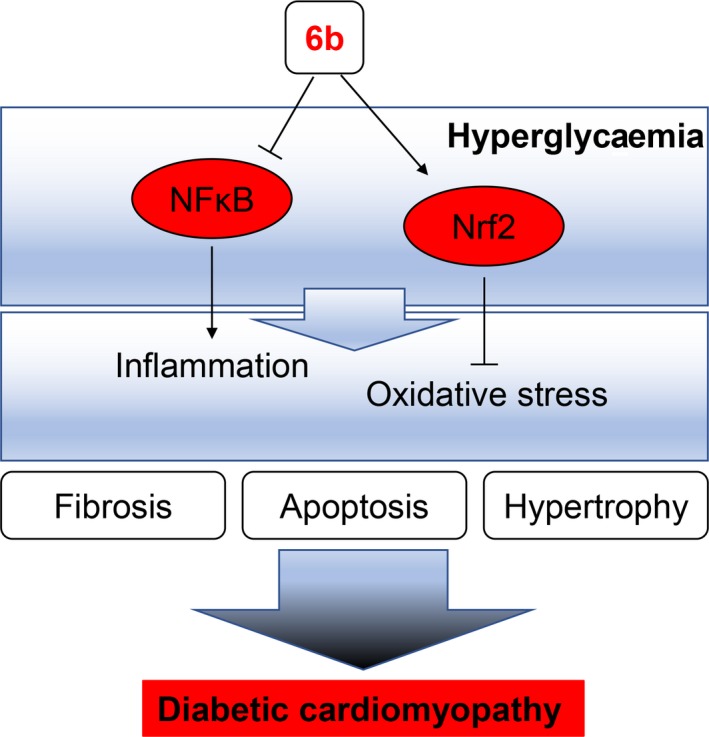
A schematic illustration for the prevention of compound 6b from diabetes/HG‐induced injury in cardiomyocytes and heart.

A number of studies had reported that resveratrol and chalcones have potential activities in inhibition of inflammatory 11, 18 and oxidant stress 19, 20. Previously, our group had synthesized Aza resveratrol–chalcone compounds and evaluated their biochemical properties in LPS‐stimulated macrophages. The results showed that compound 6b has the highest potency in inhibiting LPS‐induced IL‐6 release. Moreover, 6b exhibited protection against LPS‐induced acute lung injury *in vivo* 13. In this study, we showed that 6b potently prevent the progression of DCM by inhibiting inflammation response and alleviating oxidative stress, Figure 8.

Chronic and sustained inflammation may be one of the major reasons that hyperglycaemia causes structural and functional alterations in the cardiac muscle 21. Unbalanced glycemic and fatty acid metabolism increase the expression of inflammatory cytokines (IL‐1β, IL‐6, TNF‐α and TGF‐β1), and cell adhesion molecules (ICAM‐1 and VCAM‐1 in the heart 22, 23, 24. Anti‐inflammatory strategy had been demonstrated beneficial towards cardiac function 12, 25. In our study, 6b decreased the expression of inflammatory cytokines and adhesion molecules induced by HG/diabetes both *in vitro* and *in vivo*. This salutary effect of 6b was mediated by blocking the nucleus translocation of NF‐κB, a key transcription factor that controls inflammation.

Besides cardiac inflammation, oxidative stress has also been implicated in all stages of DCM, from cardiac hypertrophy to fibrosis, contractile dysfunction and heart failure 26. Oxidative stress, demonstrated by elevated levels of ROS and RNS, was mainly originated from mitochondrial dysfunction caused by metabolic disorders. Genes involved in regulating oxidative stress have been revealed to be either directly or indirectly regulated by Nrf2 14. Nrf2 is normally distributed in the cytoplasm. Upon activation by stress conditions, it will translocate into the nucleus to initiate the expression of antioxidative proteins including HO‐1 and NQO‐1 14. Nrf2 activation has been shown to be involved in the protective effects against DCM. Here, we have found that 6b attenuated oxidative stress mechanistic through up‐regulating and activating of Nrf2.

Inflammation and oxidative stress together contribute to cardiac cell dysfunction and apoptosis 27, 28. Therefore, we have investigated the effects of 6b on cell morphology and apoptosis in hyperglycaemia‐induced *in vitro* and *in vivo*. In STZ‐induced diabetic mice, treatment of 6b resulted in reduced expression of pro‐apoptotic protein Bax and the level of cleaved PARP and increased expression of antiapoptotic protein Bcl‐2 levels. Subsequently, the number of apoptotic cells assessed by TUNEL staining was less in 6b treatment group compared to the vehicle control group. These results indicate that inhibition of cardiac cell apoptosis may be one of the crucial mechanisms by which 6b protects against DCM. On the other hand, LV dysfunctional is an early hallmark of DCM 29. However, the pathogenesis of LV dysfunction in the diabetic heart has not been fully clarified. One of the explanations lies in the overall changes of cardiac morphology and histology, including fibrosis and hypertrophy, which can also be mediated by inflammation and oxidative stress 30, 31. We observed that while HG and hyperglycaemia induce the expression of pro‐fibrotic markers (collagen I, TGF‐β and CTGF) and cardiac hypertrophic markers (ANP, BNP and MyHC), 6b significantly attenuated the up‐regulation of these fibrotic and hypertrophic biomarkers. Moreover, the subsequent cardiac morphological changes, including interstitial collagen deposition, increased LV mass and cardiac contractile dysfunction, were all ameliorated by 6b preconditioning.

The current work convincingly demonstrated that hyperglycaemia‐induced cardiac inflammation and oxidative stress contribute to the disease progression of DCM. It also provided a deeper understanding of the regulatory role of NF‐κB and Nrf2 in hyperglycaemia‐induced cardiac injury. The findings of present study reinforced the defensive role of 6b against inflammation, oxidative stress, apoptosis, hypertrophy and fibrosis both *in vitro* and *in vivo* and further suggested that NF‐κB and Nrf2 may be promising therapeutic targets for treating DCM. It should be noted that many factors such as the type of treatment, metabolic characteristics, lipid profile and other individual differences may affect the DCM.

Even though a tremendous amount of effort has been put on the novel therapies for the prevention and treatment of DCM, there is still an unmet medical needs in this field. New molecule entities derived from natural compounds such as 6b exhibiting both antioxidant and anti‐inflammatory properties may attract more attention for the treatment of DCM. For future work, we will continue more sophisticated mechanistic investigation of 6b as a potential antidiabetic drug candidate.

## Conclusion

Inflammation and oxidative stress are involved in the pathogenesis of DCM. Present study demonstrated the preventive role of 6b against inflammation, oxidative stress, hypertrophy, fibrosis and apoptosis in DCM mechanistically through blocking NF‐κB nucleus translocation and activating Nrf2 both *in vitro* and *in vivo*. Therefore, the Aza resveratrol–chalcone derivative could be a potential therapeutic drug in treating DCM. Moreover, NF‐κB and Nrf2 may be important therapeutic targets for diabetic complications.

## Conflicts of interest

All the authors declare no competing financial interest.

## Supporting information


**Table S1.** Primers used for real‐time qPCR assay.
**Figure S1.** The effects of 6b treatment on the profiles of body weight, blood glucose and insulin in STZ‐induced diabetic mice.
**Figure S2.** The effects of 6b on H9c2 viability.
**Figure S3.** The effects of 6b on cardiomyocyte size in STZ‐induced diabetic mice.
**Figure S4.** 6b attenuates NF‐κB nucleus translocation.
