# Supplementary material for: An Aza resveratrol–chalcone derivative 6b protects mice against diabetic cardiomyopathy by alleviating inflammation and oxidative stress
Source: J Cell Mol Med. 2018 Jan 12;22(3):1931–43. doi: 10.1111/jcmm.13477 (PMC5824376; doi:10.1111/jcmm.13477)
Supplement: Supplementary file 1 — Table S1. Primers used for real‐time qPCR assay. Figure S1. The effects of 6b treatment on the profiles of body weight, blood glucose and insulin in STZ‐induced diabetic mice. Figure S2. The effects of 6b on H9c2 viability. Figure S3. The effects of 6b on cardiomyocyte size in STZ‐induced diabetic mice. Figure S4. 6b attenuates NF‐κB nucleus translocation. [file JCMM-22-1931-s001.doc]

**An Aza resveratrol-chalcone derivative 6b Protects Mice against Diabetic Cardiomyopathy by Alleviating Inflammation and Oxidative Stress**

Shengban You^1,2,#^, Wenbing Jiang^3,#^, Jianchang Qian^1,#^, Hailing Zhang^4^, Shiju Ye^2^, Taiwei Chen^2^, Zheng Xu^1^, Jingying Wang^1^, Weijian Huang^2,*^, Guang Liang^1,*^

**Supplementary Figures and Legends**

| Gene | Species | Primers(FW) | Primers(RW) |
| --- | --- | --- | --- |
| ANP | Mouse | AACCTGCTAGACCACCTGGA | TGCTTTTCAAGAGGGCAGAT |
| BNP | Mouse | GTCAGTCGTTTGGGCTGTAAC | AGACCCAGGCAGAGTCAGAA |
| TGF-β | Mouse | TGACGTCACTGGAGTTGTACGG | GGTTCATGTCATGGATGGTGC |
| Collagen1 | Mouse | TGGCCTTGGAGGAAACTTTG | CTTGGAAACCTTGTGGACCAG |
| IL-6 | Mouse | GAGGATACCACTCCCAACAGACC | AAGTGCATCATCGTTGTTCATACA |
| IL-1β | Mouse | ACTCCTTAGTCCTCGGCCA | CCATCAGAGGCAAGGAGGAA |
| Nrf2 | Mouse | TTTTCCATTCCCGAATTACAGT | AGGAGATCGATGAGTAAAAATGGT |
| β-actin | Mouse | CCGTGAAAAGATGACCCAGA | TACGACCAGAGGCATACAG |
| Collagen1 | Rat | GACATCCCTGAAGTCAGCTGC | TCCCTTGGGTCCCTCGAC |
| TGF-β | Rat | GCAACAACGCAATCTATGAC | CCTGTATTCCGTCTCCTT |
| C-TGF | Rat | GCCTGTTCCAAGACCTGT | TGTCCGGATGCACTTTTTGC |
| MyHC | Rat | CGAGTCCCAGGTCAACAAG | AGGCTCTTTCTGCTGGACA |
| VCAM-1 | Rat | TTTGCAAGAAAAGCCAACATGAAAG | TCTCCAACAGTTCAGACGTTAGC |
| TNF-α | Rat | TACTCCCAGGTTCTCTTCAAGG | GGAGGCTGACTTTCTCCTGGTA |
| IL-6 | Rat | GAGTTGTGCAATGGCAATTC | ACTCCAGAAGACCAGAGCAG |
| TNF-α | Rat | TACTCCCAGGTTCTCTTCAAGG | GGAGGCTGACTTTCTCCTGGTA |
| Nrf2 | Rat | ACTGTCCCCAGCCCAGAGGC | CCAGGCGGTGGGTCTCCGTA |
| NQO-1 | Rat | ACCTTGCTTTCCATCACCAC | CAAAGGCGAAAACTGAAAGC |
| HO-1 | Rat | TCTATCGTGCTCGCATGAAC | CAGCTCCTCAAACAGCTCAA |
| β-actin | Rat | AAGTCCCTCACCCTCCCAAAAG | AAGCAATGCTGTCACCTTCCC |

**Table S1:** Primers used for real-time qPCR assay.

**
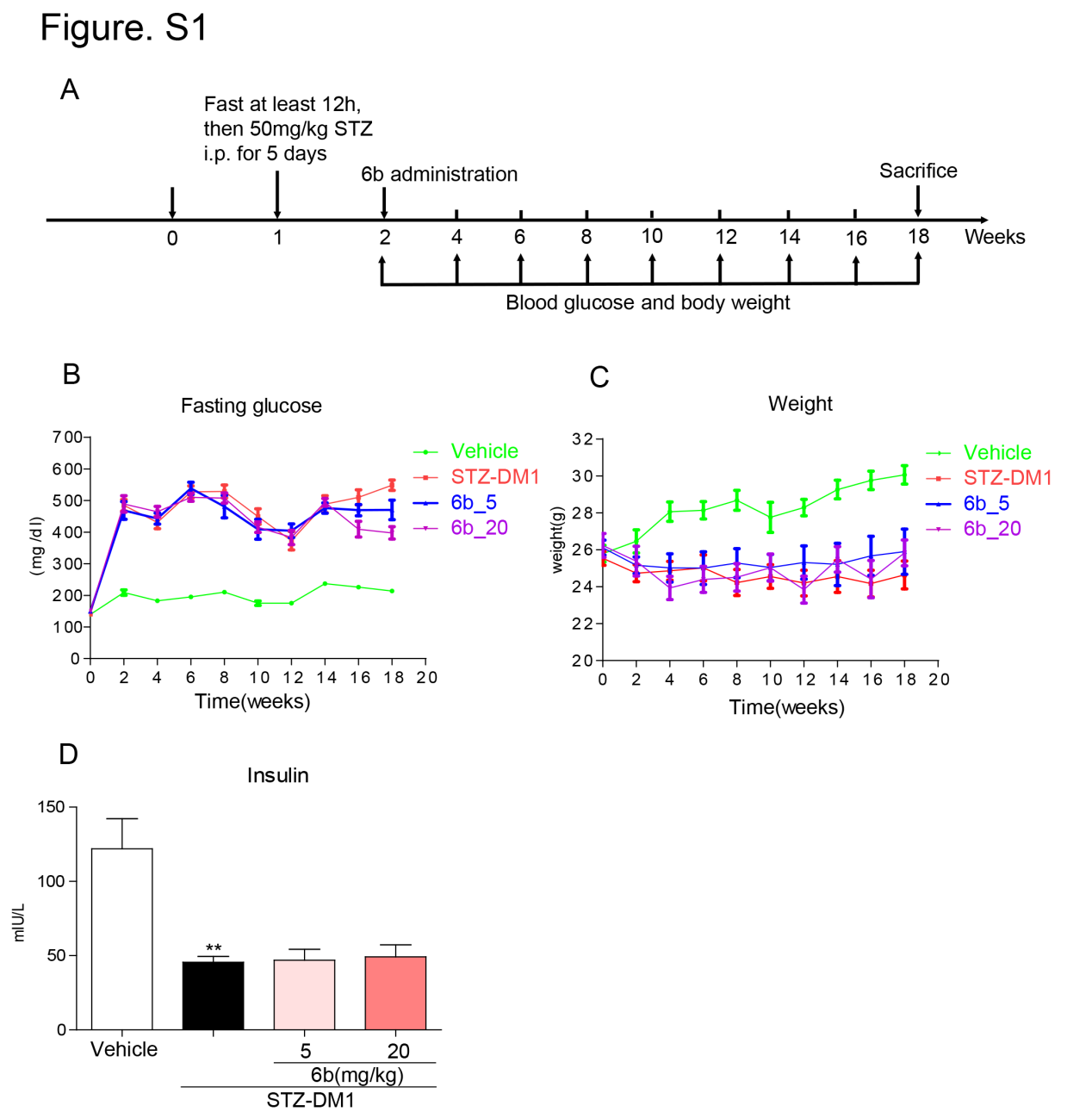
**

**Supplementary Figure 1.** The effects of 6b treatment on the profiles of body weight, blood glucose and insulin in STZ-induced diabetic mice. A. Protocol for the STZ induction of diabetes and the treatment regimen of 6b. B and C. The curves of blood glucose and body weight of mice were plotted. D. Blood samples were collected as mice being sacrificed. Serum insulin were subjected to ELISA assay as described in Methods. **, *p* < 0.01 *vs* Vehicle.


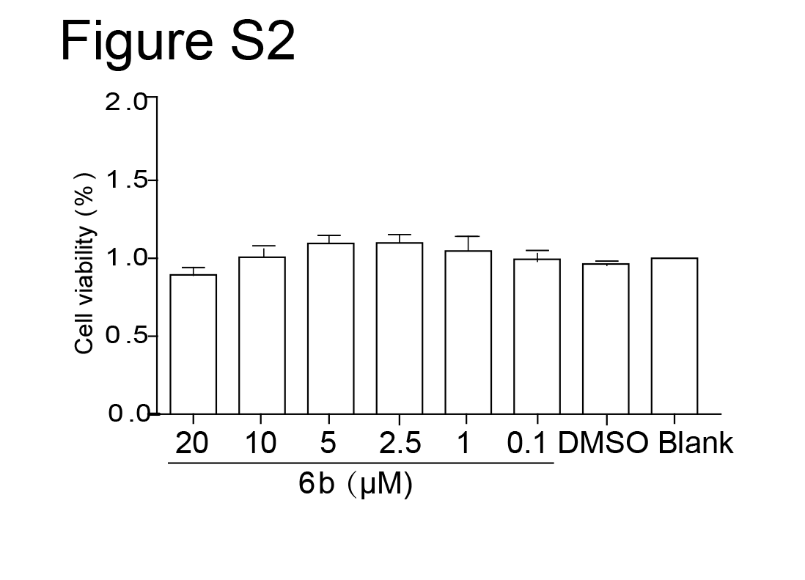


**Supplement Figure 2.** The effects of 6b on H9c2 viability. H9c2 cells treated with 6b at indicated dose, and subjected to MTT assay after 48 h treatment as described in Methods. Blank, the well with no DMSO.


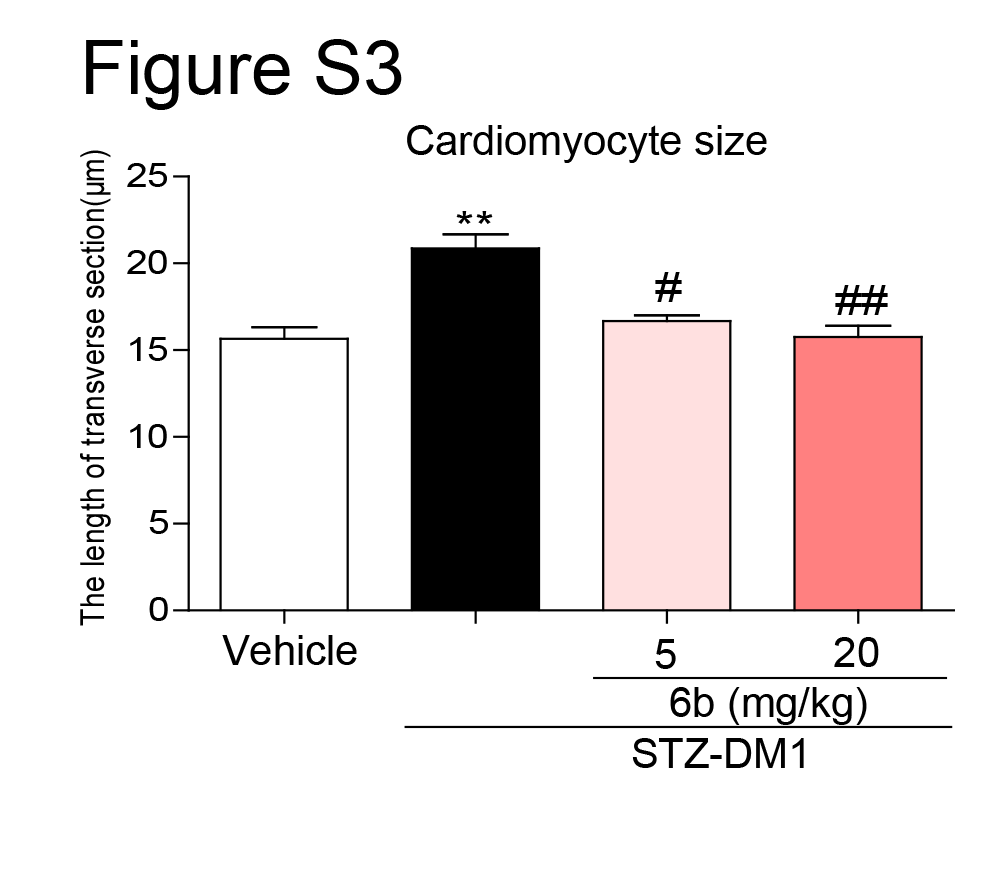


**Supplementary Figure 3.** The effects of 6b on cardiomyocyte size in STZ-induced diabetic mice. Heart tissues were collected after mice being sacrificed. Cardiomyocyte transverse cross-sectional area of tissues were measured. Normalized values are plotted. **, *p* < 0.01 *vs* Vehicle; #, *p* < 0.05, ##, *p* < 0.01 *vs* STZ-DM1.


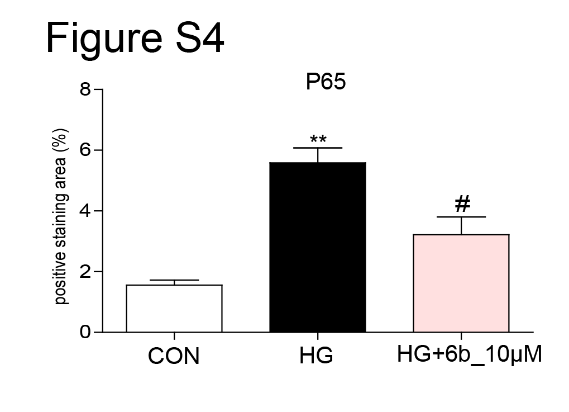


**Supplement Figure 4.** 6b attenuates NF-κB nucleus translocation. H9c2 cells were pretreated with 6b as indicated for 1 h, and then were incubated with HG (33 mM) for following 8 h. Cells were subjected to immunofluorescence assay as described in Methods. Images were acquired and quantified. **, *p* < 0.01 *vs* CON; #, *p* < 0.05 *vs* STZ-DM1.
